# Cost-effectiveness of exercise therapy versus general practitioner care for osteoarthritis of the hip: design of a randomised clinical trial

**DOI:** 10.1186/1471-2474-12-232

**Published:** 2011-10-12

**Authors:** Pauline P van Es, Pim AJ Luijsterburg, Joost Dekker, Marc A Koopmanschap, Arthur M Bohnen, Jan AN Verhaar, Bart W Koes, Sita MA Bierma-Zeinstra

**Affiliations:** 1 Erasmus MC, University Medical Center, Department of General Practice, PO Box 2040, 3000 CA Rotterdam, the Netherlands; 2 VU Medical Center, EMGO Institute, PO Box 7057, 1007 MB Amsterdam, the Netherlands; 3 Institute for Medical Technology Assessment, Erasmus University Rotterdam, PO Box 1738, 3000 DR Rotterdam, the Netherlands; 4 Erasmus MC, University Medical Center, Department of Orthopaedics, PO Box 2040, 3000 CA Rotterdam, the Netherlands

## Abstract

**Background:**

Osteoarthritis (OA) is the most common joint disease, causing pain and functional impairments. According to international guidelines, exercise therapy has a short-term effect in reducing pain/functional impairments in knee OA and is therefore also generally recommended for hip OA. Because of its high prevalence and clinical implications, OA is associated with considerable (healthcare) costs. However, studies evaluating cost-effectiveness of common exercise therapy in hip OA are lacking. Therefore, this randomised controlled trial is designed to investigate the cost-effectiveness of exercise therapy in conjunction with the general practitioner's (GP) care, compared to GP care alone, for patients with hip OA.

**Methods/Design:**

Patients aged ≥ 45 years with OA of the hip, who consulted the GP during the past year for hip complaints and who comply with the American College of Rheumatology criteria, are included. Patients are randomly assigned to either exercise therapy in addition to GP care, or to GP care alone. Exercise therapy consists of (maximally) 12 treatment sessions with a physiotherapist, and home exercises. These are followed by three additional treatment sessions in the 5th, 7th and 9th month after the first treatment session. GP care consists of usual care for hip OA, such as general advice or prescribing pain medication. Primary outcomes are hip pain and hip-related activity limitations (measured with the Hip disability Osteoarthritis Outcome Score [HOOS]), direct costs, and productivity costs (measured with the PROductivity and DISease Questionnaire). These parameters are measured at baseline, at 6 weeks, and at 3, 6, 9 and 12 months follow-up. To detect a 25% clinical difference in the HOOS pain score, with a power of 80% and an alpha 5%, 210 patients are required. Data are analysed according to the intention-to-treat principle. Effectiveness is evaluated using linear regression models with repeated measurements. An incremental cost-effectiveness analysis and an incremental cost-utility analysis will also be performed.

**Discussion:**

The results of this trial will provide insight into the cost-effectiveness of adding exercise therapy to GPs' care in the treatment of OA of the hip. This trial is registered in the Dutch trial registry http://www.trialregister.nl: trial number NTR1462.

## Background

Osteoarthritis (OA) is the most common joint disease. It is a chronic condition causing pain and disability of especially hip and knee joints [1,2]. On January 1 2007 about 238, 000 persons were registered in a general practitioner's (GP) database in the Netherlands because of symptoms of hip OA [3]. Incidence and prevalence will increase in the coming years due to ageing of the population. The rapid increase in persons aged ≥ 55 years in Western countries implies that OA is becoming a public healthcare problem with increasing healthcare costs [1,4].

In addition to the societal influence, OA affects daily activities and quality of life. The GP is the initial caregiver involved, in many cases providing the patient with advice and medication over many years. The GP may also refer to exercise therapy. International guidelines recommend exercise therapy as part of the treatment of OA [5-7]. The Osteoarthritis Research Society International (OARSI) [7] reviewed 51 treatment modalities that were investigated in studies published 2006-2009, in which exercise therapy was shown to reduce pain and improve physical functioning in patients with knee OA [8]. Studies investigating OA have often recruited both hip OA and knee OA patients [2,9]. However, because few hip patients were recruited and no joint-specific data were reported, recommendations for the treatment of hip and/or knee OA are mainly based on knee OA studies; less evidence for the effectiveness of exercise therapy on hip OA symptoms is available. This underlines the importance of obtaining more data on the effects of exercise therapy in hip OA.

Hip OA is associated with markedly reduced lower limb muscle strength [10]. Hernandez-Molina et al. reported that the most effective therapeutic exercise involves regular aerobic activity and/or a strengthening program [11]. Their meta-analysis combining 8 trials with similar exercise strategies for hip OA, demonstrated exercise benefit with an effect size of -0.46 (95% CI -0.64, -0.28; P < 0.0001) [11]; in that meta-analysis, most exercise programs lasted maximally 12 weeks. The beneficial effects of exercise therapy seem to decline and eventually disappear [12,13]. Although it is suggested that adding booster exercise sessions to the intervention may induce long-term effects [12], few studies include such booster sessions [2,9,14].

In a comparison of three studies investigating the cost-effectiveness of exercise therapy, the reported costs per quality-adjusted life year (QALY) ranged from -$503 (cost saving) to $11.530 [7]. To gain insight into the cost-effectiveness of exercise therapy added to GP care, compared to GP care alone, additional economic evaluations alongside randomised controlled trials are needed.

Although evidence was found for the effectiveness of exercise therapy on pain and activity limitations associated with hip OA, more insight into short-term and long-term effects is needed. Because studies on the cost-effectiveness in this area of research are lacking, an economic evaluation of exercise therapy in OA is also needed.

Therefore, the present research questions are:

1) What is the cost-effectiveness of exercise therapy added to GP care, compared to GP care alone, in patients with OA of the hip?

2) What is the effectiveness of exercise therapy added to GP care, compared to GP care alone, in patients with OA of the hip?

## Methods/Design

### Study design

**Figure 1:**
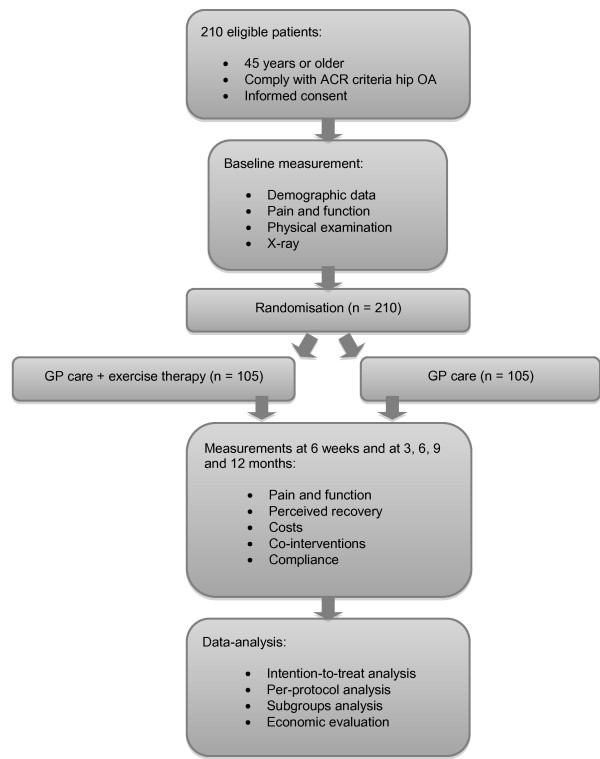
**Flowchart of the study**.

This study is a multi-centre randomised controlled trial with a parallel group design. Follow-up lasts for 12 months. Figure 1 presents the flowchart of the study. This study is approved by the Erasmus MC Medical Ethical committee.

### Population

The target population consists of patients suffering from hip OA, 45 years and older, who have consulted their GP during the past year regarding hip complaints. Patients are identified via searches in the patient registries of the participating GPs.

### Patient recruitment

**Figure 2:**
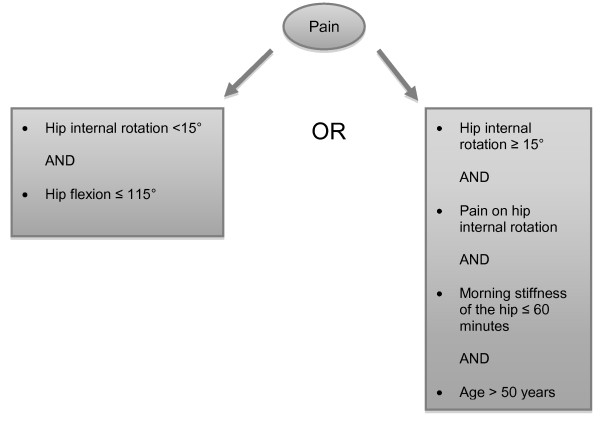
**Clinical ACR criteria Hip**.

Participating GPs in the area of Rotterdam will invite patients with hip OA who visited them during the past year for hip complaints and who comply with the clinical American College of Rheumatology (ACR) criteria [15] for hip OA (Figure 2). After receiving a positive patient's response, researchers screen for eligibility. If eligible and patients' written informed consent is obtained, the baseline measurement is conducted (Figure 1).

### Inclusion/exclusion criteria

Patients are eligible if they consulted the GP for a new episode of non-traumatic hip complaints during the past year, are 45 years or older, comply with the clinical ACR criteria for hip OA, and complete the informed consent procedure. Researchers determine whether the patient complies with the ACR criteria by conducting a physical examination at baseline.

Patients are excluded if they meet one of six exclusion criteria: 1) has received exercise therapy in the past 3 months; 2) hip pain score < 2 on an 11-point numeric rating scale (NRS) (0 means no pain); 3) hip surgery in the past or on the waiting list for hip surgery; 4) severe disabling co-morbidity that disallows receiving exercise therapy; 5) insufficient comprehension of the Dutch language; and/or 6) mentally incapable of participation.

### Randomisation

The randomisation procedure is based on a computer-generated randomisation list provided by a person independent to the randomisation procedure. Based on this list, opaque, sealed, sequentially numbered, non-reusable envelopes are prepared for the researchers to open after the baseline measurement. To prevent unequal group sizes, block randomisation is used with random blocks of 4, 6 and 8 patients.

Randomisation is performed by the researcher who conducts the baseline measurement. After performing all baseline measurements, the researcher opens a sealed envelope in the presence of the patient. The contents of the envelope are revealed to both the researcher and the patient.

The randomisation procedure assigns patients to a group receiving exercise therapy in conjunction with GP care, or to a group receiving GP care alone.

### Blinding

Due to the study design and the use of questionnaires, GPs, patients and therapists cannot be blinded to the intervention.

### Interventions

Patients are randomised to a group receiving exercise therapy in conjunction with GP care (intervention group), or to a group receiving GP care alone (control group). Patients in the intervention group are referred to a physiotherapist who is trained to administer exercise therapy according to the study protocol.

#### Physiotherapy

The exercise therapy supervised by physiotherapists consists of (maximally) 12 evenly spread treatment sessions during 3 months. If treatment goals are reached before the 12^th ^treatment session, physiotherapists are allowed to stop the treatment before the 12^th ^treatment session. Nevertheless, the first block of treatment sessions is followed by three treatment sessions in the 5^th^, 7^th ^and 9^th ^month. One treatment session lasts 25-30 minutes.

A consensus meeting was organised for the participating physiotherapists. During this meeting, the exercise therapy protocol based on the Dutch guideline for physiotherapy [16] was discussed and trained in detail. A booklet with exercises was created to stimulate treatment adherence regarding home exercises. A selection of these exercises is shown in Additional file 1.

Physiotherapists are instructed to administer exercise therapy as they usually do in patients with hip OA, taking into account the limitations set by the study protocol. The protocol states that passive treatment forms (such as manual therapy, massage and joint traction) are not allowed. A treatment session should always include giving advice to the patient about OA and lifestyle adaptations. Furthermore, the instruction and evaluation of home exercises are considered to be key elements. Physiotherapists are allowed to administer exercises and home exercises of their choice. As physiotherapists mainly follow their own approach, treatments may differ slightly between therapists. Some patients will receive individual therapy, while others will exercise in groups. Methods, treatment goals, intensity and frequency are tailored to the patient's needs. Using standardised forms, physiotherapists register the duration of treatment sessions, number of treatment sessions, individual treatment goals, adherence to home exercises, and any possible violation of the protocol.

The general aims of exercise therapy are to improve functioning, increase levels of activity, and to encourage an adequate way of dealing with the complaints. Individual aims may consist of reducing pain, improving muscle strength, active joint stability, coordination, joint mobility, endurance and/or the performance of activities.

#### GP care

GP care is provided as usual in daily practice by the patient's own GP. Patients in either group are allowed unrestricted visits to their GP for hip complaints as required. The GP may provide education and counselling and/or prescribe pain medication. Furthermore, the GP may refer the patient to an orthopaedic surgeon or request additional diagnostic tests or examinations.

After admission to the study, patients in both groups receive an identical brochure with general information and advice. The topics covered include the diagnosis and prognosis of hip OA, as well as advice on daily activities, the use of (walking) aids, medical treatment and exercise. The information in the brochure was developed by the Dutch College of General Practitioners [17].

### Study parameters

The measurements at baseline and at 12 months follow-up consist of a physical examination in conjunction with questionnaires. All other measurements consist of questionnaires only. At baseline a pelvic X-ray (anteroposterior) is taken.

Main study parameters are hip pain and hip-related activity limitations (measured with the Hip disability Osteoarthritis Outcome Score (HOOS) [18,19]), direct costs and productivity costs (measured with the PROductivity and DISease Questionnaire (PRODISQ) [20]). These outcomes are obtained at baseline, at 6 weeks, and at 3, 6, 9 and 12 months follow-up (Table 1). The HOOS questionnaire is an extension of the Western Ontario McMaster Universities (WOMAC) Osteoarthritis Index [21]); WOMAC pain and function scores are calculated based on HOOS scores.

**Table 1:** Outcome measures

	Baseline	6 w	3 m	6 m	9 m	12 m
**Primary outcome measures**						
Pain and function (HOOS)	X	X	X	X	X	X
Direct costs and productivity costs (PRODISQ)	X	X	X	X	X	X
**Secondary outcome measures**						
Hip pain (NRS)	X	X	X	X	X	X
Hip pain (ICOAP)	X	X	X	X	X	X
Perceived recovery	X	X	X	X	X	X
Quality of life (EuroQol EQ-5D)	X	X	X	X	X	X
Functional mobility (Timed Up and Go test)	X					X
Range of motion (physical examination)	X					X
Demographic data	X					
Radiographic osteoarthritis parameters (X-ray)	X					
Compliance to assigned treatment		X	X	X	X	X
Co-interventions	X	X	X	X	X	X

w: weeks

m: months

HOOS: Hip disability Osteoarthritis Outcome Score questionnaire

PRODISQ: PROductivity and DISease Questionnaire

NRS: Numeric Rating Scale

ICOAP: Intermittent and Constant Osteoarthritis Pain questionnaire

The following secondary study parameters are measured with questionnaires at baseline, at 6 weeks, and at 3, 6, 9 and 12 months follow-up:

- Hip pain, measured with an NRS score (0-10 scale) [22]

- Hip pain, measured with the Intermittent and Constant Osteoarthritis Pain (ICOAP) questionnaire [23,24]

- Perceived Recovery, measured on a 7-point Likert scale [25]

- Quality of life, measured with the EuroQol, EQ-5D [26].

In addition, activity limitations are measured with the Timed Up and Go test [27]. Range of motion of both hips is also obtained. These tests are performed at baseline and at 12 months follow-up.

In this study, the following demographic data are collected:

- Age, gender, height, weight, education, duration of complaints, previous hip pain, co-morbidity.

- Radiographic OA parameters, obtained with X-ray (anteroposterior pelvic).

- Active and passive activity limitations, and range of motion of the hip (obtained by the physical examination at baseline and at 12 months follow-up).

- Compliance to assigned treatment.

- Co-interventions, including referral of patients in the control group to physiotherapy.

### Sample size calculation

It is expected that 25% of patients assigned to the group that receives GP care only will be referred to physiotherapy (cross-over). Also, a 10% loss to follow-up is expected. To detect a 25% clinical difference in the WOMAC pain score (mean 4.83, SD 2.25) after 12 months with two-tailed testing, a power of 80% and an alpha 5%, 210 patients are required [28].

### Statistical analysis

#### Effectiveness

Success of randomisation and distribution of outcome measures is assessed before actual analyses are performed. Data collected during 12 months follow-up are analysed according to the intention-to-treat principle using linear regression models with repeated measurements. Risk differences are calculated for HOOS outcome scores of the intervention and the control group. Risk differences are adjusted for possible differences between groups concerning prognostic factors.

In addition, risk differences are analysed per protocol. In the intervention group, patients receiving < 60% (≈ 12) of the maximum number of exercise therapy sessions are not considered. In the control group, crossovers are not considered.

Subgroup analyses are planned for the following variables: age (45-65 years vs. > 65 years), pain intensity (NRS score ≥ 3), education, radiographic severity and co-morbidity (low-back pain or knee pain). Because of insufficient power the subgroups are analysed for explorative purpose only.

#### Economic evaluation

In an incremental cost-effectiveness analysis, societal costs during the first year are compared with the primary outcome measure (HOOS and WOMAC, averaged over the first year) and perceived recovery (7-point Likert scale). For comparison with a wider range of interventions an incremental cost-utility analysis is conducted, comparing the incremental societal costs of the intervention per patient during the first year and the incremental quality of life in years/days, as obtained from the EuroQol (QALY during the first year). Utility values of the Dutch public for EuroQol health states are applied [29]. The costs per unit of medical consumption and the costs per hour productivity lost are estimated using updated information from the Dutch Manual for economic evaluation of health care [30]. The friction cost method is used to calculate the productivity costs according to the Dutch guidelines. Costs are reported for the year 2009. Using non-parametric bootstrapping (randomly drawing 2500 observations with replacement from the patient sample), the degree of uncertainty for costs and health effects, and the cost-utility ratio, is depicted in a so-called cost-effectiveness plane.

In addition an acceptability curve is drawn, which indicates the probability that the intervention has lower incremental costs per QALY gained than various thresholds for the maximum willingness to pay for an extra QALY. An attempt is made to model differences in effectiveness and costs beyond the time horizon of the study itself, by extrapolating the success rates of exercise therapy, GP consultations, required surgery due to hip OA, and absenteeism. Here, time horizons of 2 and 5 years are used with discounting of costs by 4% per year and health effects by 1.5% per year, according to the pharmacoeconomic guidelines of the Dutch Health Care Insurance Board [31].

## Discussion

Patient recruitment is expected to be finished by end 2011. Based on a 12-month follow-up, the results are expected early 2013. The results of this trial will provide insight into the cost-effectiveness of adding exercise therapy to GPs' care in the treatment of OA of the hip.

## Competing interests

The authors declare that they have no competing interests.

## Authors' contributions

PAJL, JD, MAK, JANV, BWK and SMABZ conceived the study, developed the trial design and contributed to writing the article. AMB contributed to writing the article. PPVE participated in completing the study design, drafted the article and will conduct the research. All authors have read and approved the final version of the article.

## Pre-publication history

The pre-publication history for this paper can be accessed here:

http://www.biomedcentral.com/1471-2474/12/232/prepub

## Supplementary Material

Additional file 1 **Selection of home exercises**. A selection of home exercises in the booklet that is given to patients in the exercise therapy group. Click here for file
