# Supplementary material for: Cost-effectiveness of exercise therapy versus general practitioner care for osteoarthritis of the hip: design of a randomised clinical trial
Source: BMC Musculoskelet Disord. 2011 Oct 12;12:232. doi: 10.1186/1471-2474-12-232 (PMC3198764; doi:10.1186/1471-2474-12-232)

Appendix

1. Position yourself lying on your side. Lift your leg up. Ensure that your hip and knee are kept straight and your foot points outwards. Switch sides.

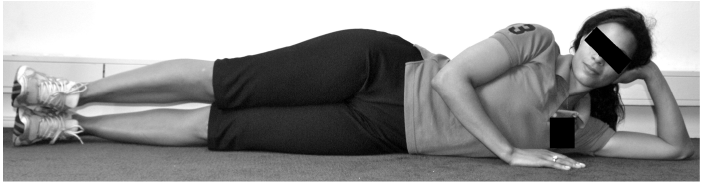

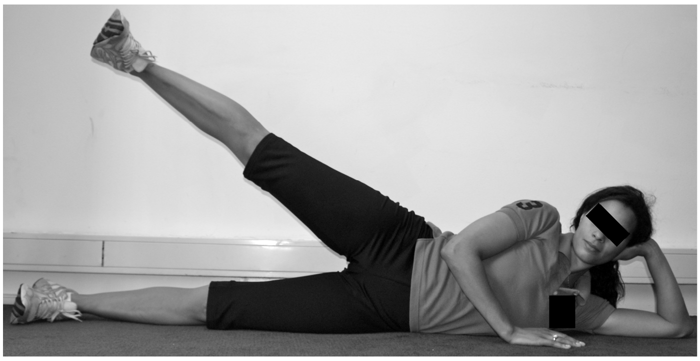

2. Position yourself with your heels and shoulders against a wall. Pull your knee to your chest.

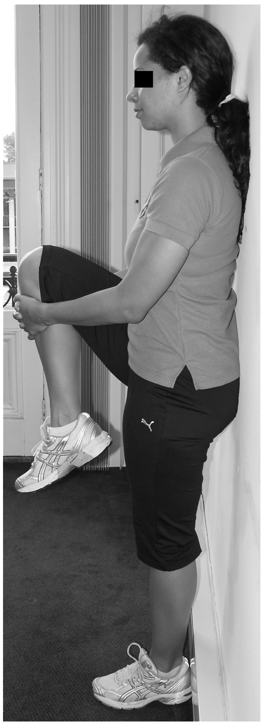

3. Position yourself on hands and knees. Extend your right leg, keeping your back as straight as possible. Ensure that your knee is kept straight and your foot points downwards.

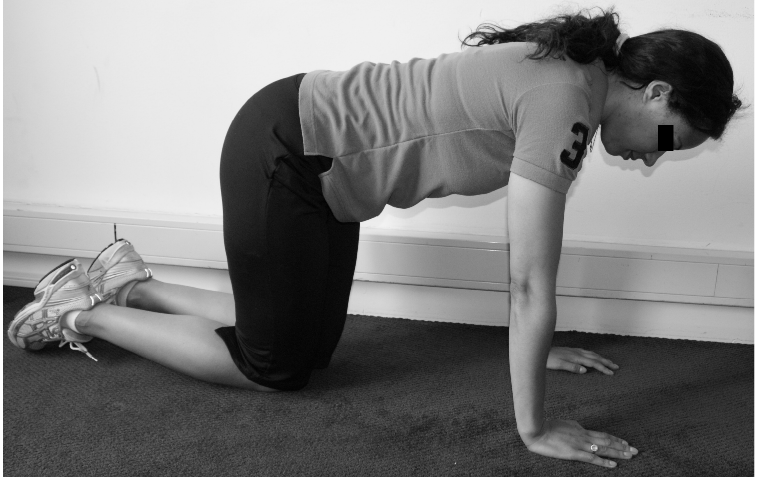

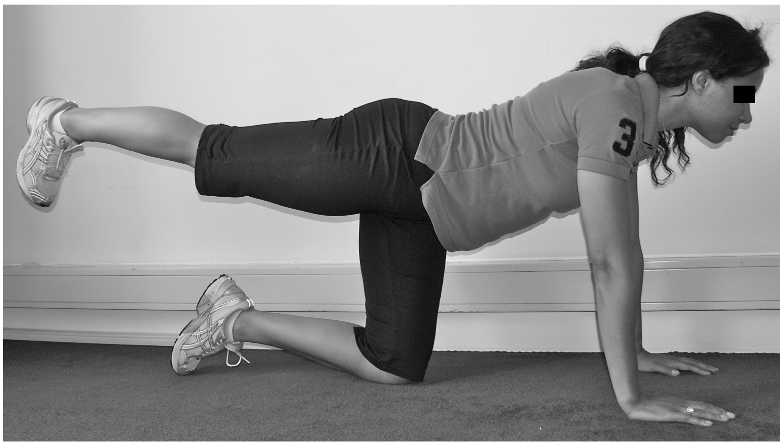

4. Position yourself at the foot of the stairs. Place one foot on the first step and bend your anterior knee, bringing your weight forward. Ensure your torso is upright, your posterior knee is straight and your posterior heel touches the floor.

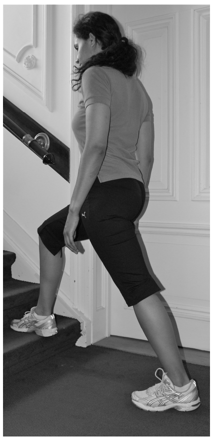

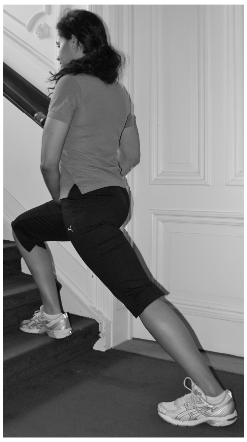

Supplement: Additional file 1 — Selection of home exercises. A selection of home exercises in the booklet that is given to patients in the exercise therapy group. [file 1471-2474-12-232-S1.PDF]
